# Impact of mobility reduction on COVID-19 mortality: absence of evidence might be due to methodological issues

**DOI:** 10.1038/s41598-021-02461-2

**Published:** 2021-12-07

**Authors:** Gideon Meyerowitz‐Katz, Lonni Besançon, Antoine Flahault, Raphael Wimmer

**Affiliations:** 1 Faculty of Health and Society, University of Wollongong, Wollongong, Australia; 2 Faculty of Information Technology, Monash University, Clayton, Australia; 3 University of Geneva, Geneva, Switzerland; 4 University of Regensburg, Regensburg, Germany

**Keywords:** Epidemiology, Epidemiology, Health policy

**arising from**: R. F. Savaris et al.; *Scientific Reports* 10.1038/s41598-021-84092-1 (2021).

## Introduction

Identifying the impact of Non-Pharmaceutical Interventions (NPIs) against COVID-19 has been an important topic throughout the pandemic. It is, in many ways, one of the most difficult scientific challenges facing the research community, as these interventions are often implemented in parallel, and it is challenging to disentangle how various methods of avoiding infection may have impacted case and death rates across the world.

To this vital question, we read with interest the paper written by Savaris et al. entitled “Stay-at-home policy is a case of exceptional fallacy: an internet-based ecological study”^1^.

The authors found no evidence that COVID-19 deaths were reduced by more people staying at home in early 2020. However, we believe that several key deficiencies within the methodology make their conclusions unsupported.

Savaris et al. developed a custom approach to detect whether changes in mobility and COVID-19-related mortality in a country are associated: for each country in their dataset, and for each week between 2020-02-16 and 2020-08-22, they calculated the relative change in residential mobility and the relative change in COVID-19-related deaths. For every possible pair of countries A and B, they then subtracted the mobility/mortality time series of country A from the mobility/mortality time series of country B. Finally, the authors calculated the regression for “difference in change in mobility between the countries” versus ”difference in change of mortality between the countries”. They interpret a statistically significant correlation (i.e., the slope is not zero) as evidence of an association between mobility and mortality. As only about 5% of comparisons are statistically significant at p < 0.05 and only 1.6% passed further statistical tests—Savaris et al. argue that there is little evidence that staying home reduces the number of deaths/million.

## Unclear suitability of the chosen approach

In our opinion, the chosen statistical approach may not be able to detect an effect of staying at home (or changes in mobility) on mortality at all.

The approach does not intuitively make sense to us as it intentionally removes potentially meaningful information from the raw data. For example, the temporal order of the weekly mobility/mortality changes is not taken into account. This means that—by design—the approach cannot determine causality, i.e., whether changes in mobility affect mortality or changes in mortality affect mobility. It also seems unusual that the inherent delay^2–5^ between changes in mobility and their potential effect on mortality is not considered in this approach at all. Furthermore, only the weekly *change* in mobility/mortality is analyzed, ignoring the absolute magnitude of both values. Góes^6^ argues that the approach is actually mathematically incapable of determining whether a correlation between mobility and mortality exists.

Given these observations, we see reason to assume that the novel approach proposed by Savaris et al. is not actually suitable for investigating the question at hand. The authors did not demonstrate that their approach would actually be able to detect an effect if it existed.

## Failure to replicate

Thankfully, the authors published source code and data that allow for replicating their analysis. We could reproduce the results shown in the second figure of their paper using the provided dataset and the Jupyter notebook. It seems that the p-values in the second figure of their paper have not been corrected for multiple comparisons, so the legend might be misleading. In the following, we do not adjust for multiple comparisons, either.

To determine whether the approach could actually detect an effect of changes in mobility on mortality, we conducted four experiments using the code in Savaris’ Jupyter notebook.

**Figure 1:**
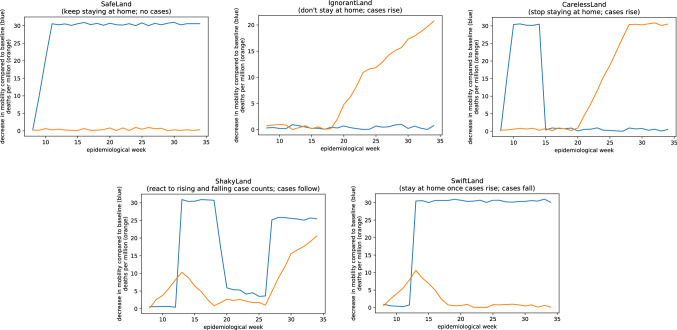
Five hypothetical countries where there is an obvious effect of changes in mobility on mortality.

**Figure 2:**
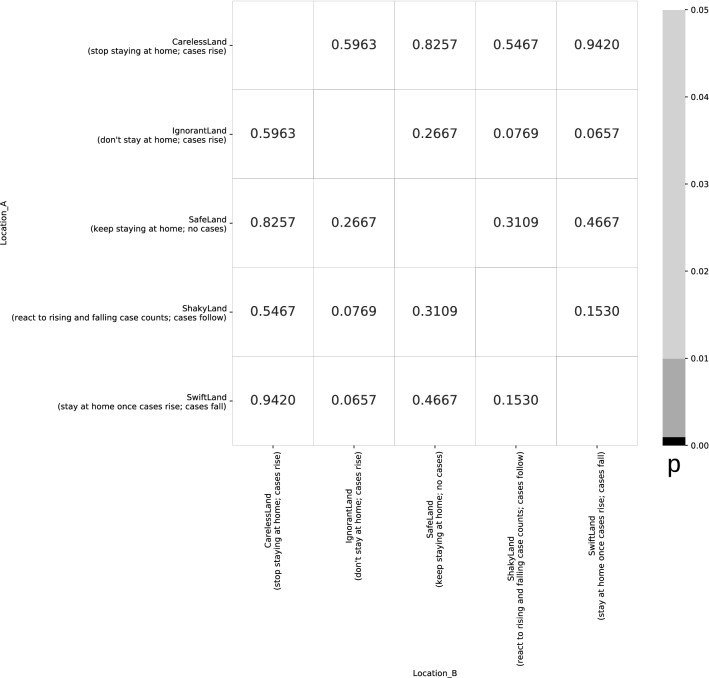
Heatmap of comparisons between model countries showing p-values calculated using the approach by Savaris et al. Grey/black cells would denote comparisons where the regression gives a p-value of < 0.05.

First, we constructed a data set with five hypothetical countries where changes in mobility (or the lack thereof) had an obvious effect on mortality (Fig. 1). When running this dataset through the Jupyter notebook, none of the comparisons were statistically significant at p < 0.05 (Fig. 2). According to the authors’ criteria, this would mean that even for our handcrafted dataset, no effect of mobility on mortality could be found.

**Figure 3:**
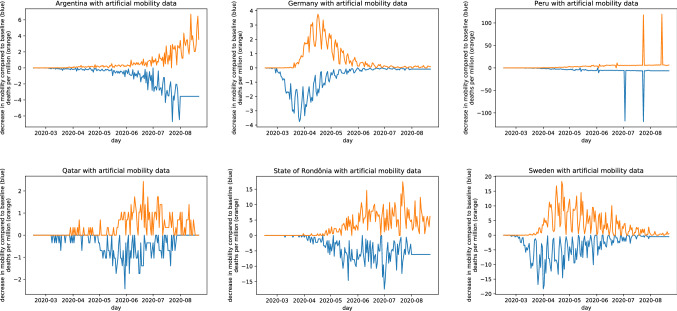
A sample of six countries (of 87 altogether) with original mortality data and artificial mobility data. In all these countries, a change in mobility precedes an equally strong change in mortality—which would be clear evidence of a correlation between mobility and mortality. In the original dataset analysed by Savaris et al., the reported mortality for some countries (e.g., Peru) is obviously not correct. To allow comparison of our results to those by Savaris et al., we did not correct such errors.

To address concerns that the approach might just be unsuitable for artificial datasets that don’t resemble real data, we created another dataset for which a working algorithm should detect an effect of mobility on mortality: for each of the 87 countries in the original dataset, we kept the mortality time series but generated a new mobility time series. This was done by inverting each value in the mortality dataset and shifting the data to the left by 3 weeks. Missing values at the end of the time series were set to the last valid value. This resulted in a mobility time series where each change in mobility would “result” in a change in mortality exactly three weeks later (Fig. 3).

**Figure 4:**
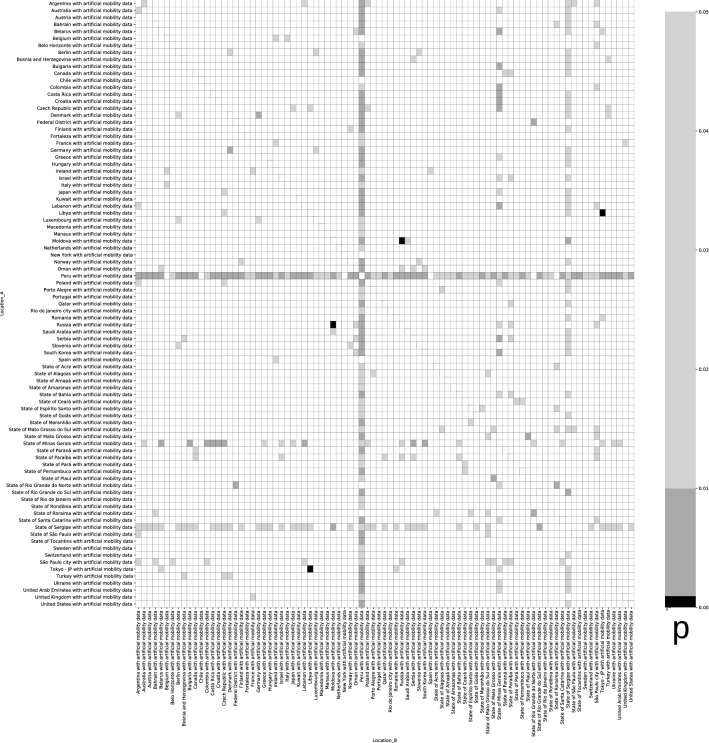
Heatmap of comparisons between countries from the original dataset with artificial, matching mobility data, showing p-values calculated using the approach by Savaris et al. Grey/black cells denote comparisons where the regression gives a p-value of < 0.05 (414 of 7482, 5.533%).The horizontal and vertical lines of dark-grey cells are caused by comparisons with the obviously erroneous dataset for Peru.

Running this dataset through the notebook resulted in 414 of 7482 comparisons (5.533%) being statistically significant (Fig. 4). This is quite precisely the expected result if there was no association at all in the dataset^8^—indeed, the expected result if the code could not detect any effect would be 5% of values at p < 0.05 by definition. However, a majority of “significant comparisons” were caused by the obviously erroneous dataset from Peru. Following the reasoning by Savaris et al., all of the comparisons should have been statistically significant.

**Figure 5:**
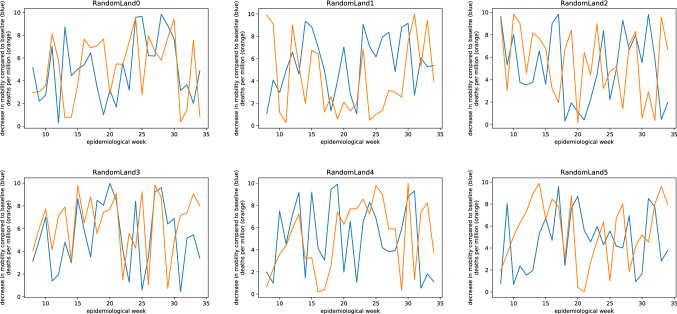
A sample of six countries (of 87 altogether) with random mortality and mobility data.

**Figure 6:**
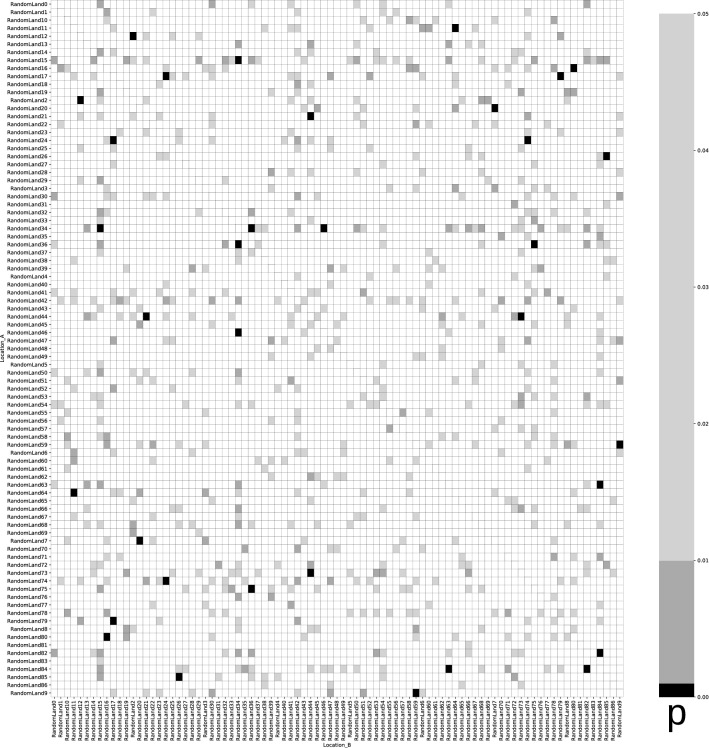
Heatmap of comparisons between countries with random mobility/mortality data. Grey/black cells denote comparisons where the regression gives a p-value of < 0.05 (742 of 7482, 9.917%).

As a third experiment, we applied the algorithm by Savaris et al. to a dataset of 87 artificial countries with mobility/mortality data generated by a pseudo-random number generator (Fig. 5). This resulted in 742 of 7482 (9.917%) statistically significant comparisons at p < 0.05 (Fig. 6). That means that random data seems to provide better evidence for an effect of mobility on mortality than both datasets that we designed to demonstrate such an effect.

**Figure 7:**
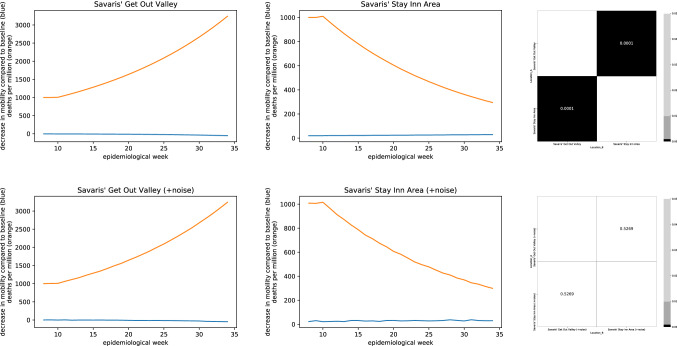
Time series and heatmaps for two sample countries provided by Savaris et al. (top) and the same dataset with a tiny bit of noise added (bottom). Whereas applying the method by Savaris et al. to the original time series results in all comparisons being statistically significant, adding noise makes the comparisons non-significant.

As we were unable to identify datasets for which the approach by Savaris et al. would actually result in statistically significant comparisons, we asked the authors for an example for which their method would demonstrate an effect of lockdowns. They sent a dataset with two hypothetical countries where lockdowns are indeed effective in reducing mortality and staying open results in a rising number of deaths. For this dataset, the notebook indeed outputs a p-value of < 0.0001 (Fig. 7, top). However, adding a small amount of random noise to the dataset resulted in a non-significant regression again (Fig. 7, bottom).

To us, the outcomes of these experiments indicate that the proposed method (a) cannot reliably detect an effect of mobility on mortality even if it should exist, (b) cannot distinguish meaningful data from random data, and (c) is highly susceptible to noise in the data.

Overall, this seems to make the approach unsuitable for the task at hand.

## Google mobility data

While Google’s mobility data is opaque and dimensionless it is not unreasonable to use such data as rightfully pointed out by the authors who cite previous work^9,10^. However, unlike prior works, the authors used only the “residential” category of the mobility data. Residential changes, as noted by Google^11^, are the least likely to show any impact when behaviour changes, since people already spend a significant amount of time at home in normal circumstances. This does not mean that residential mobility scores are always low, but that they will always be the lowest of the mobility metrics and thus most likely to result in a null comparison. Moreover, they are the least likely to *change* on a weekly basis, and since the author’s model only takes into account the change, this would inherently bias the analyses towards a null result.

## Ecological fallacy

Ecological studies of this kind are also geared towards null findings. As one example, the country of Australia is included as a single unit of analysis. However, during the time period of the study, Australia had a unique situation, with a single state (Victoria) locking down for months while the rest of the country opened up. At the country level this difference becomes almost negligible, with an increase of < 10% in “residential” mobility across Australia. However, in Victoria alone, the increase exceeded 20% for this entire period. Any comparisons to Australia as a whole during the Victorian lockdown are hence unreliable as a measure of the key metrics of interest, and while this is perhaps most significant for Australia it is clearly not limited to a single nation. Russia, the United States, and many other places suffer from the exact same issue. This limitation is noted in the discussion, but it is not just a notation but a fundamental limitation that makes the final numbers largely arbitrary. In addition, it should be noted that the death numbers from Belarus are overtly implausible, and the numbers from Peru contain obvious reporting errors. Both countries should have been excluded from the analysis.

## Confounding by indication

Finally, including only countries with more than 100 overall COVID-19 deaths in the analysis excludes countries such as New Zealand, Singapore, and Vietnam that had success with rapid, strict measures. This introduces a bias towards countries without effective measures and therefore increases the chance that no difference between countries can be found^5,12^.

## Discussion

While the question of whether NPIs can influence COVID-19 deaths is of great importance, the issues we have highlighted seriously weaken the conclusions made by the authors. That they found no statistically significant differences between regions on these metrics may simply be a function of their chosen methodology and the inherent limitations of the mobility dataset, and might have little to do with the matter at hand. It appears likely that the methodology could not detect an effect of staying at home on mortality even if one were to exist.

## Data Availability

The source code and datasets used for our attempt at replication can be found on our GitHub repository^7^.
